# Supplementary material for: An origami paper-based nanoformulated immunosensor detects picograms of VEGF-C per milliliter of blood
Source: Commun Biol. 2021 Jan 26;4:121. doi: 10.1038/s42003-020-01607-8 (PMC7838172; doi:10.1038/s42003-020-01607-8)
Supplement: Supplementary file 2 — Description of Additional Supplementary File [file 42003_2020_1607_MOESM2_ESM.pdf]

Description of additional supplementary file

File name: Supplementary Data 1

Description: Source data for main figures 2 – 6.
