# Supplementary material for: An origami paper-based nanoformulated immunosensor detects picograms of VEGF-C per milliliter of blood
Source: Commun Biol. 2021 Jan 26;4:121. doi: 10.1038/s42003-020-01607-8 (PMC7838172; doi:10.1038/s42003-020-01607-8)
Supplement: Supplementary file 4 — Reporting Summary [file 42003_2020_1607_MOESM4_ESM.pdf]

nature research

Corresponding author(s): Xinxia Cai

Last updated by author(s): Oct 10, 2020

Reporting Summary

Nature Research wishes to improve the reproducibility of the work that we publish. This form provides structure for consistency and transparency in reporting. For further information on Nature Research policies, see our Editorial Policies and the Editorial Policy Checklist.

Statistics

For all statistical analyses, confirm that the following items are present in the figure legend, table legend, main text, or Methods section.

n/a	Confirmed	
☐	☒	The exact sample size (n) for each experimental group/condition, given as a discrete number and unit of measurement
☐	☒	A statement on whether measurements were taken from distinct samples or whether the same sample was measured repeatedly
☐	☒	The statistical test(s) used AND whether they are one- or two-sided<br>*Only common tests should be described solely by name; describe more complex techniques in the Methods section.*
☐	☒	A description of all covariates tested
☐	☒	A description of any assumptions or corrections, such as tests of normality and adjustment for multiple comparisons
☐	☒	A full description of the statistical parameters including central tendency (e.g. means) or other basic estimates (e.g. regression coefficient) AND variation (e.g. standard deviation) or associated estimates of uncertainty (e.g. confidence intervals)
☐	☒	For null hypothesis testing, the test statistic (e.g. F, t, r) with confidence intervals, effect sizes, degrees of freedom and P value noted<br>*Give P values as exact values whenever suitable.*
☐	☒	For Bayesian analysis, information on the choice of priors and Markov chain Monte Carlo settings
☐	☒	For hierarchical and complex designs, identification of the appropriate level for tests and full reporting of outcomes
☐	☒	Estimates of effect sizes (e.g. Cohen's d, Pearson's r), indicating how they were calculated

Our web collection on statistics for biologists contains articles on many of the points above.

Software and code

Policy information about availability of computer code

Data collection	Nova 2.1
Data analysis	OriginPro

For manuscripts utilizing custom algorithms or software that are central to the research but not yet described in published literature, software must be made available to editors and reviewers. We strongly encourage code deposition in a community repository (e.g. GitHub). See the Nature Research guidelines for submitting code & software for further information.

Data

Policy information about availability of data

All manuscripts must include a data availability statement. This statement should provide the following information, where applicable:

- Accession codes, unique identifiers, or web links for publicly available datasets
- A list of figures that have associated raw data
- A description of any restrictions on data availability

All data needed to evaluate the conclusions in the paper are present in the paper and/or the Supplementary Materials. Additional data related to this paper are available from the corresponding author upon reasonable request.

Field-specific reporting

Life sciences study design

All studies must disclose on these points even when the disclosure is negative.

Sample size	According to previous experience, if we want to evaluate the performance of the sensor, we need to test at least three times for each concentration of substance, and draw the error bar. Therefore, we use the sensor proposed in this paper to detect the VEGF-C of each concentration for three times, and the results are as described in the paper. Then we detected more than ten clinical serum samples, in order to verify the reliability of the sensor.
Data exclusions	No data were excluded from the analyses
Replication	To improve the reproducibility of the immunosensor, we firstly optimized the fabrication process of the paper-based device to make sure that the unmodified electrodes exhibited the same performances. Moreover, the electrochemical properties are mainly based on the nanomaterials. As a result, we strictly controlled the synthetic process, including the mixing time, mixing ratio, and the concentrations of each materials.
Randomization	The allocation was random
Blinding	The investigators were blinded to group allocation during data collection and analysis

Reporting for specific materials, systems and methods

We require information from authors about some types of materials, experimental systems and methods used in many studies. Here, indicate whether each material, system or method listed is relevant to your study. If you are not sure if a list item applies to your research, read the appropriate section before selecting a response.

Materials & experimental systems

n/a	Involved in the study	
☐	☒	Antibodies
☒	☐	Eukaryotic cell lines
☒	☐	Palaeontology and archaeology
☒	☐	Animals and other organisms
☒	☐	Human research participants
☐	☒	Clinical data
☒	☐	Dual use research of concern

Methods

n/a	Involved in the study	
☒	☐	ChIP-seq
☒	☐	Flow cytometry
☒	☐	MRI-based neuroimaging

Antibodies

Antibodies used	Anti-VEGF-C antibody came from Beijing Biolink Biotechnology
Validation	Anti-VEGF-C antibody was obtained from Beijing Biolink Biotechnology. The number of the product is 2445.

Clinical data

Policy information about clinical studies

All manuscripts should comply with the ICMJEguidelines for publication of clinical research and a completedCONSORT checklist must be included with all submissions.

Clinical trial registration	All clinical serum samples were provided by Peking University Cancer Hospital & Institute. All clinical serum samples have been reviewed by the ethics committee and can be used for experiments. (2015KT04)
Study protocol	The clinical samples had already been tested in the central lab of the hospital using a commercial-available Roche Cobos E602 electrochemiluminescence apparatus. The clinical serum samples were detected directly with our sensor without any dilution and the analytical results were compared with the reference values provided by the hospital.
Data collection	Incubation was required for the binding of antigen and corresponding antibody before detection and it would last for about 15 min. All electrochemical experiments were carried out in an Autolab potentiostat in PBS solution (pH=7.4). The DPV curves were recorded in the range of −0.5 V to 0.3 V, with 5 mV step potential, 0.025 s modulation time and 0.5 s interval time.
Outcomes	According to the obtained linear fitting curve of peak current against VEGF-C concentration for clinical serum samples, we could calculate its corresponding concentrations of VEGF-C.
